# Artemlavanins A and B from *Artemisia lavandulaefolia* and Their Cytotoxicity Against Hepatic Stellate Cell Line LX2

**DOI:** 10.1007/s13659-020-00254-0

**Published:** 2020-06-24

**Authors:** Cheng Shen, Xiao-Yan Huang, Chang-An Geng, Tian-Ze Li, Shuang Tang, Li-Hua Su, Zhen Gao, Xue-Mei Zhang, Jing Hu, Ji-Jun Chen

**Affiliations:** 1 State Key Laboratory of Phytochemistry and Plant Resources in West China, Kunming Institute of Botany, Chinese Academy of Sciences, Kunming, 650201 People’s Republic of China; 2 Yunnan Key Laboratory of Natural Medicinal Chemistry, Kunming, 650201 People’s Republic of China; 3 University of Chinese Academy of Sciences, Beijing, 100049 People’s Republic of China

**Keywords:** *Artemisia lavandulaefolia*, Sesquiterpenoids, Artemlavanins, Cytotoxicity, HSC-LX2

## Abstract

**Abstract:**

Two new sesquiterpenoids, artemlavanins A (**1**) and B (**3**), together with fifteen known compounds (**2** and **4−17**) were isolated from the EtOH extract of *Artemisia lavandulaefolia*. The structures of new compounds were elucidated by extensive spectroscopic analyses (HRESIMS, 1D and 2D NMR) and ECD calculations. Compound **1** was a sesquiterpenoid lactone possessing a rearranged eudesmane skeleton; compounds **2–5**, **6–8**, **9** and **10–12** belonged to the eudesmane, guaiane, oppositane and farnesane sesquiterpenoids, respectively; compounds **13–17** were the phenyl derivatives with a 4-hydroxyacetophenone moiety. Twelve compounds (**1–3**, **5–7**, **10–12**, **14**, **15** and **17**) displayed cytotoxicity against hepatic stellate cell line LX2 (HSC-LX2) with IC_50_ values ranging from 35.1 to 370.3 *μ*M. Compounds **2**, **7**, **10–12** and **17** exhibited the stronger cytotoxicity than silybin (IC_50_, 169.6 *μ*M) with IC_50_ values of 82.1, 35.1, 95.0, 83.8, 81.6 and 90.1 *μ*M. Compound **7** as the most active one showed significant inhibition on the deposition of human collagen type I (Col I), human hyaluronic acid (HA) and human laminin (HL) with IC_50_ values of 10.7, 24.5 and 13.3 *μ*M.

**Graphic Abstract:**

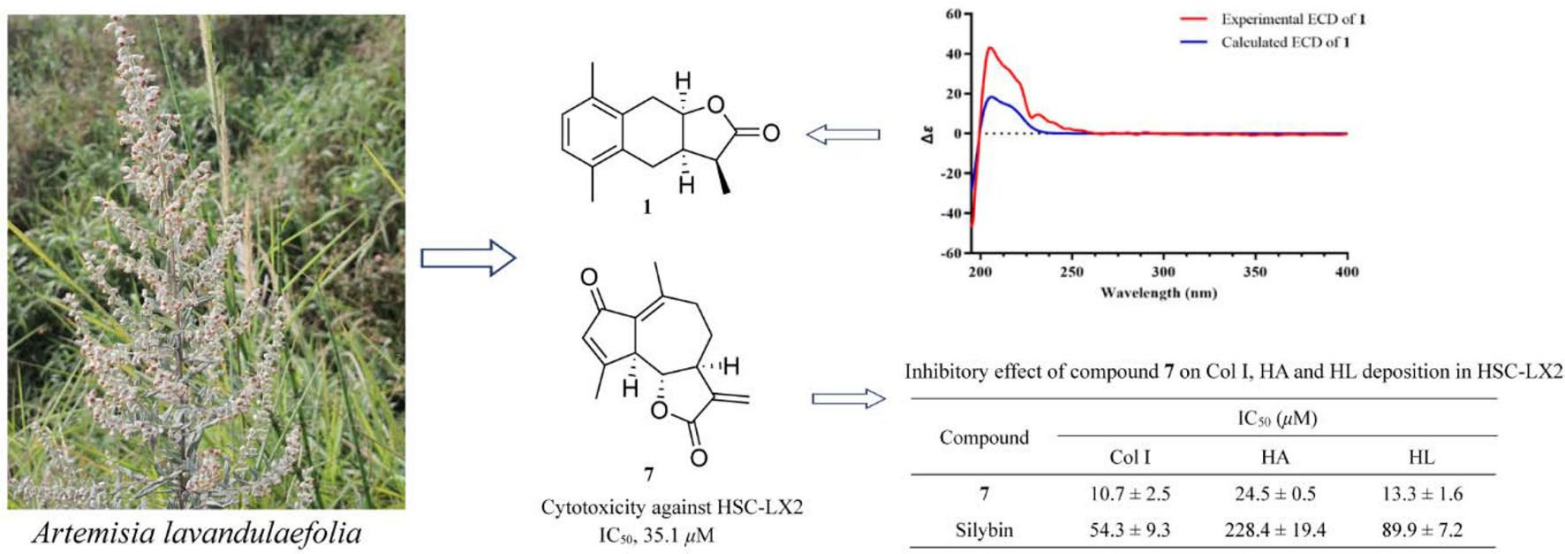

**Electronic supplementary material:**

The online version of this article (10.1007/s13659-020-00254-0) contains supplementary material, which is available to authorized users.

## Introduction

Hepatic fibrosis is characterized by the abnormal accumulation of extracellular matrix (ECM), which results from the liver diseases such as viral hepatitis and alcoholic or nonalcoholic steatohepatitis. The global prevalence of alcohol-use disorders, diabetes and hepatitis B virus were approximately 75 million, 422 million and 257 million people [1], thus increasing the risks for liver cirrhosis or cancer and leading to a significant burden for liver diseases and medical costs. Although over 20 candidate molecules for hepatic fibrosis involving the inhibition of inflammatory response, ECM production and HSCs activation have achieved positive progress in recent years, most of which are being assessed in clinical trials and none of them have been commercialized as antifibrotic drugs [2–5]. Therefore, the discovery of antifibrotic agents with high efficacy and low side effect are urgently required. Recently, a review of 60 natural products with antihepatic fibrosis activity has been reported, indicating alkaloids, polysaccharides, flavonoids, polypeptides, terpenoids and polyphenols as the active ingredients [6].

**Fig. 1:**
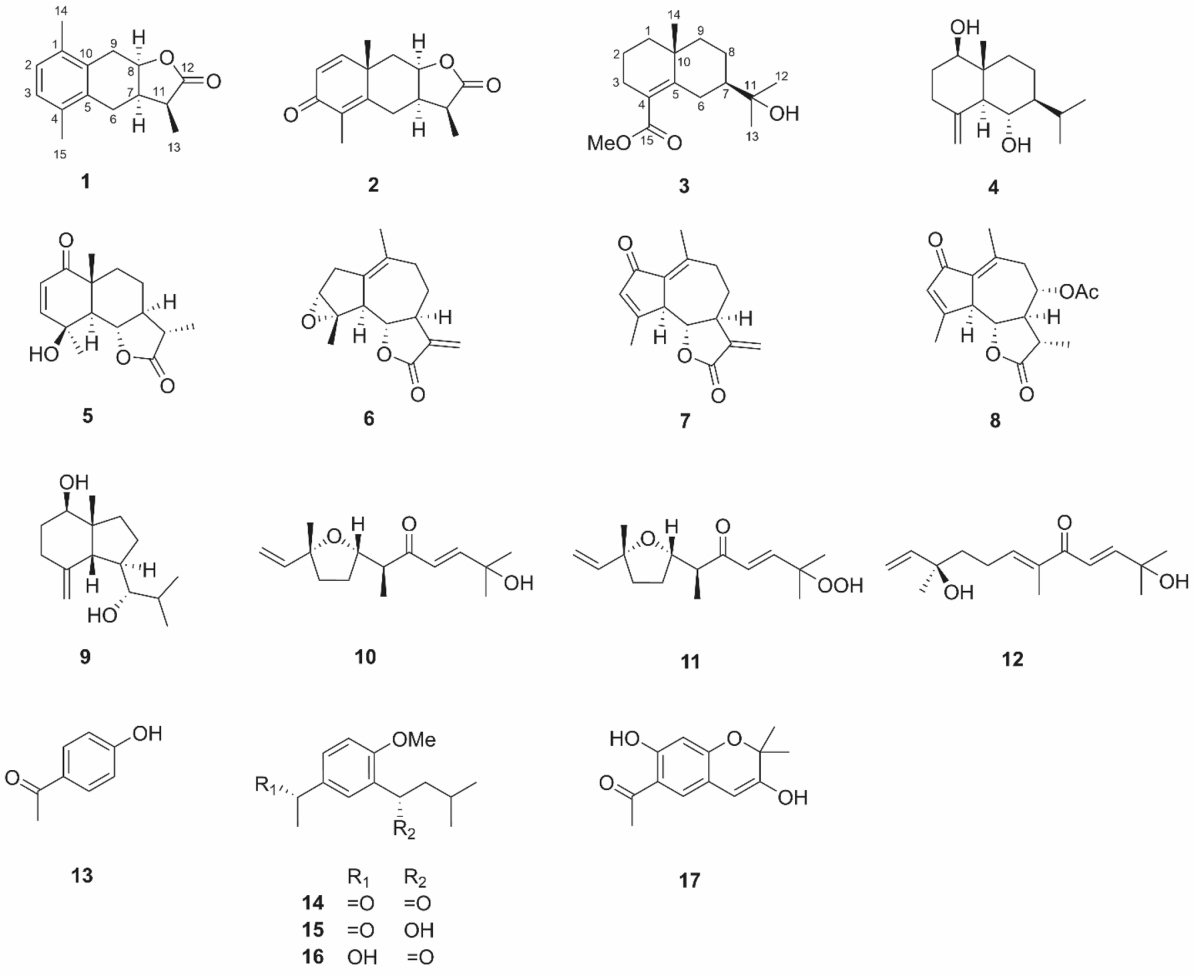
Chemical structures of compounds **1–17**

*Artemisia* is a large genus with over 300 species and belongs to the family *Asteraceae*, many of which were found to be effective for the liver diseases, such as *A. capillaris* [7, 8], *A. scoparia* [9], *A. sacrorum* [10], *A. absinthium* [11], *A. vulgaris* [12], *A. iwayomogi* [13] and *A. campestris* [14]. *A. lavandulaefolia* as a substitute of the traditional Chinese medicine *A. argyi* was used for the treatment of hemorrhage, menstruation-related symptoms, diarrhea, skin diseases, stomatitis, bronchitis and liver ailments in diverse cultures [15–17]. Phytochemical research of *A. lavandulaefolia* has led to reports on monoterpenoids [18, 19], sesquiterpenoids [19–21], triterpenoids [18, 22], steroids [18, 22], phenyl derivatives [18, 19], phenylpropanoids [18, 19, 23] and flavonoids [22, 24], as well as essential oils [17]. The present investigation on the EtOH extract of *A. lavandulaefolia* resulted artemlavanins A (**1**) and B (**3**) together with fifteen known compounds (**2** and **4–17**), which were evaluated on the hepatic stellate cell line LX2 (HSC-LX2). Herein, the isolation, structural elucidation and cytotoxicity of these compounds were reported (Fig. 1).

## Results and Discussion

**Fig. 2:**
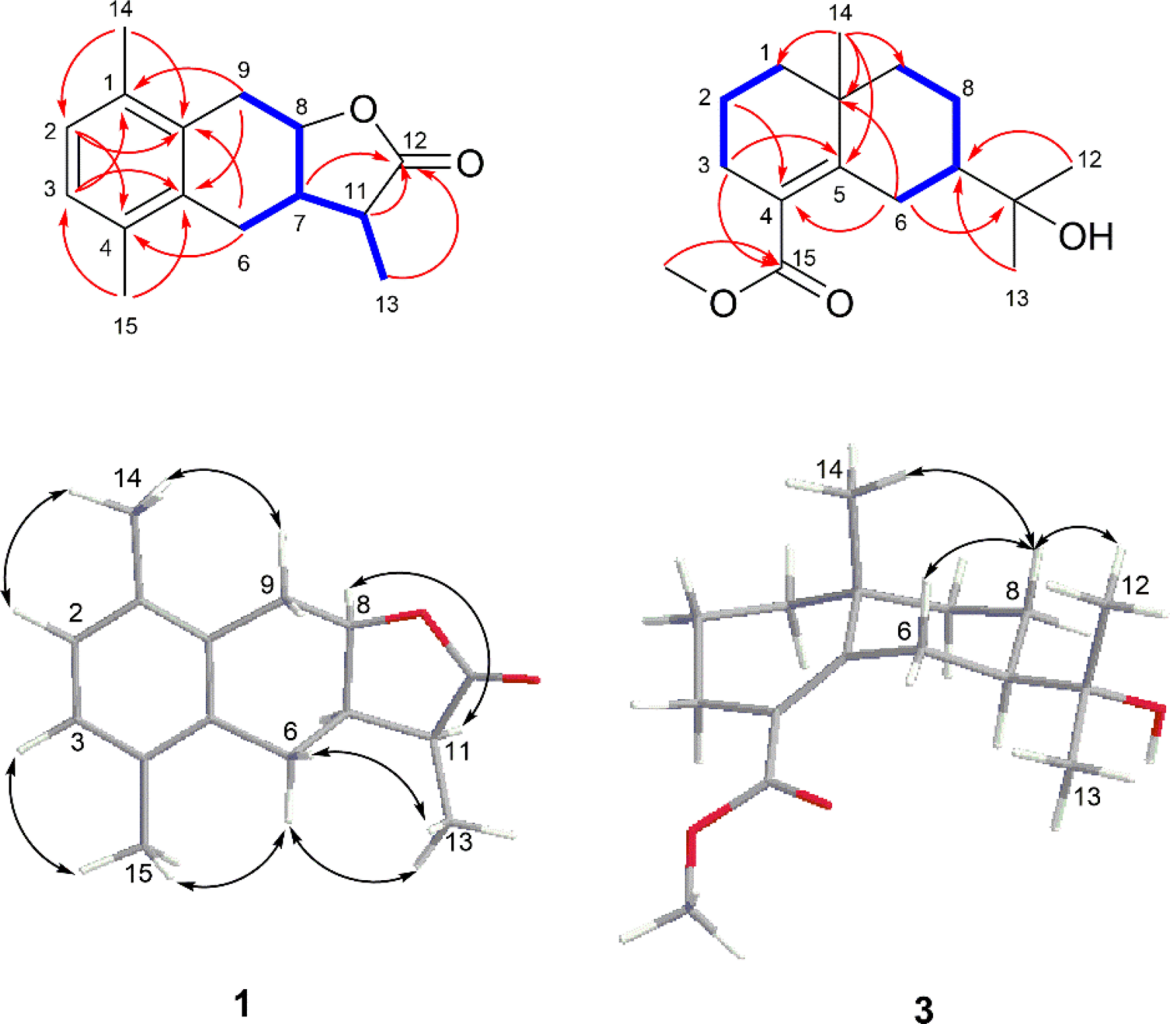
Key HMBC (red), ^1^H**–**^1^H COSY (blue) and ROESY (black) correlations of compounds **1** and **3**

**Fig. 3:**
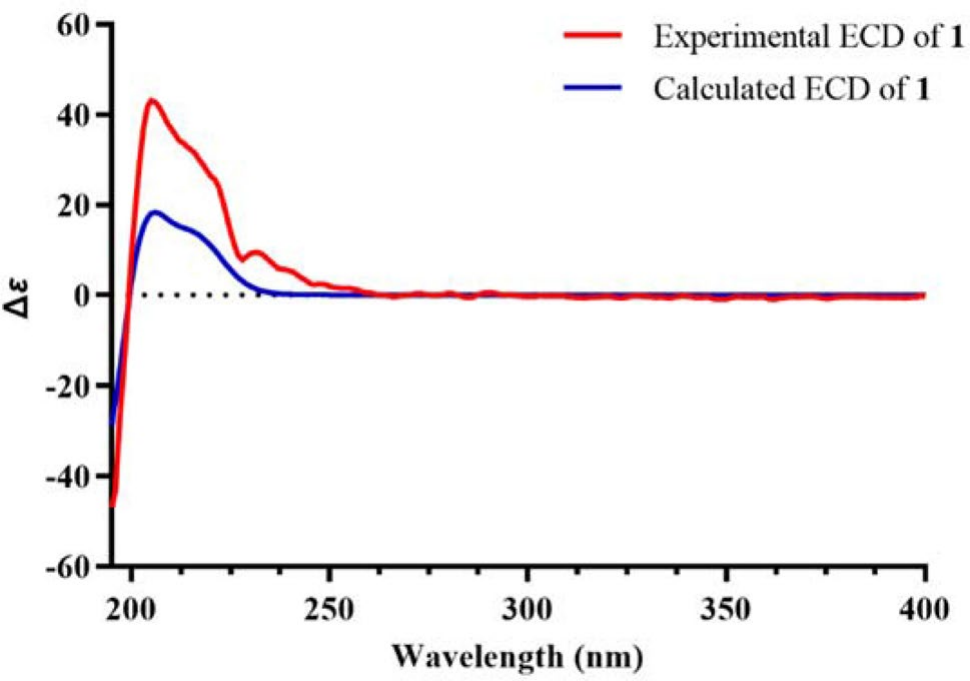
Experimental and calculated ECD spectra for compound **1** in MeOH (*σ* = 0.25, shift = ‒3 nm)

Artemlavanin A (**1**) was assigned a molecular formula of C_15_H_18_O_2_ by the (+)-HRESIMS ion at *m/z* 231.1383 [M+H]^+^ (calcd for C_15_H_19_O_2_, 231.1380), implying seven degrees of unsaturation. Its IR spectrum indicated the absorption bands for carbonyl (1752 cm^−1^) and aromatic ring (1488 cm^−1^ and 1454 cm^−1^) functionalities. The ^1^H NMR data (Table 1) displayed the presence of five methines at *δ*_H_ 6.97 (H-2 and H-3), 4.92 (H-8), 2.93 (H-11) and 2.63 (H-7), two methylenes at *δ*_H_ 3.22 (H-9a), 2.87 (H-9b), 2.73 (H-6a) and 2.38 (H-6b), and three methyl protons at *δ*_H_ 2.30 (H-15), 2.29 (H-14) and 1.28 (H-13). The ^13^C NMR (DEPT) data (Table 1) showed 15 signals for a carbonyl at *δ*_C_ 179.3, an aromatic ring at *δ*_C_ 134.8, 133.4, 132.1, 132.0 and 128.0 (× 2), three methines at *δ*_C_ 77.1, 38.3 and 38.0, two methylenes at *δ*_C_ 29.9 and 23.6, and three methyls at *δ*_C_ 19.4, 19.3 and 10.6. Two methyls were attached to C-1 and C-4 based on the HMBC correlations (Fig. 2) from H_3_-14 (*δ*_H_ 2.29) to C-2 (*δ*_C_ 128.0) and C-10 (*δ*_C_ 132.1) and from H_3_-15 (*δ*_H_ 2.30) to C-3 (*δ*_C_ 128.0) and C-5 (*δ*_C_ 134.8). The ^1^H**–**^1^H COSY cross-peaks of H-6/H-7/H-8/H-9 and H-7/H-11/H-13 together with the HMBC correlations from H_2_-6 (*δ*_H_ 2.73 and 2.38) to C-4 (*δ*_C_ 132.0) and C-10 (*δ*_C_ 132.1) and from H_2_-9 (*δ*_H_ 3.22 and 2.87) to C-1 (*δ*_H_ 133.4) and C-5 (*δ*_C_ 134.8) verified that the aromatic ring was fused with a hexatomic ring via C-10 and C-5 in compound **1**. In addition, the lactone ring was established by the HMBC correlations of H-7 (*δ*_H_ 2.63), H-11 (*δ*_H_ 2.93) and H-13 (*δ*_H_ 1.28) with C-12 (*δ*_C_ 179.3). The planar structure of compound **1** was therefore determined to be consistent with *α*-(1,4-dimethyl-7-hydroxy-5,6,7,8-tetrahydro-6-naphthyl)-propionic acid lactone as previously reported [25, 26], but the stereochemistry of this compound remained unclear. The coupling constant (*J* = 6.4 Hz) between H-7 and H-8 specified a *cis*-relationship [27, 28]. Furthermore, the ROESY correlation of H-8 with H-11 indicated that they were on the same side. Comparison of the experimental and calculated ECD spectra (Fig. 3) revealed that compound **1** was elucidated as 7*α*,11*α*H-14(10 → 1)abeo-eudesma-1,3,5(10)-trien-12,8*β*-olide.

**Table 1:** ^1^H NMR (600 MHz) and ^13^C NMR (150 MHz) data for compounds **1** and **3** in CDCl_3_ (*δ* in ppm, *J* in Hz)

No.	**1**		**3**	
	*δ*_C_	*δ*_H_	*δ*_C_	*δ*_H_
1	133.4, C	–	41.9, CH_2_	1.65, m
				1.33, m
2	128.0, CH	6.97, s	18.6, CH_2_	1.62, m
3	128.0, CH	6.97, s	28.1, CH_2_	2.31, m
				2.16, m
4	132.0, C	–	124.3, C	–
5	134.8, C	–	150.5, C	–
6	23.6, CH_2_	2.73, dd (14.9, 5.7)	28.9, CH_2_	3.00, dt (13.4, 2.5)
		2.38, dd (14.9, 9.3)		1.79, tt (13.4, 2.7)
7	38.0, CH	2.63, m	50.9, CH	1.39, m
8	77.1, CH	4.92, td (6.4, 5.1)	23.0, CH_2_	1.67, m
				1.46, m
9	29.9, CH_2_	3.22, dd (16.0, 6.4)	39.4, CH_2_	1.53, m
		2.87, dd (16.0, 5.1)		1.31, m
10	132.1, C	–	35.6, C	–
11	38.3, CH	2.93, dq (9.1, 7.4)	72.7, C	–
12	179.3, C	–	27.1, CH_3_	1.19, s
13	10.6, CH_3_	1.28, d (7.4)	27.0, CH_3_	1.19, s
14	19.4, CH_3_	2.29, s	24.9, CH_3_	1.10, s
15	19.3, CH_3_	2.30, s	171.0, C	–
−OMe	–	–	51.5, CH_3_	3.70, s

Artemlavanin B (**3**) possessed a molecular formula of C_16_H_26_O_3_ by the (+)-HRESIMS ion at *m/z* 289.1754 [M+Na]^+^ (calcd for C_16_H_26_O_3_Na, 289.1774), indicating four indices of hydrogen deficiency. The IR absorptions at 3455, 1713 and 1625 cm^‒1^ suggested the existence of hydroxy, carbonyl and double bond groups. The ^1^H NMR data (Table 1) exhibited characteristic signals for a methoxy at *δ*_H_ 3.70 (MeO-15) and three methyls at *δ*_H_ 1.10 (H_3_-14) and 1.19 (H_3_-12 and H_3_-13). The ^13^C NMR (DEPT) data (Table 1) showed 16 carbon resonances including a carbonyl at *δ*_C_ 171.0, a tetrasubstituted double bond at *δ*_C_ 150.5 and 124.3, two quaternary carbons at *δ*_C_ 72.7 and 35.6, a methine at *δ*_C_ 50.9, six methylenes at *δ*_C_ 41.9, 39.4, 28.9, 28.1, 23.0 and 18.6, three methyls at *δ*_C_ 27.1, 27.0 and 24.9, and a methoxy at *δ*_C_ 51.5. Its ^1^H NMR and ^13^C NMR spectra were similar with those of (+)-*γ-*eudesmol [29, 30], except that the methyl at C-4 in (+)-*γ-*eudesmol was replaced by a methoxycarbonyl group in compound **3**. The location of methoxycarbonyl was verified at C-4 by the HMBC correlations (Fig. 2) from MeO-15 (*δ*_C_ 3.70) and H_2_-3 (*δ*_H_ 2.31 and 2.16) to C-15 (*δ*_C_ 171.0). The relative configuration of compound **3** was identical with (+)-*γ-*eudesmol based on the ROESY correlation of H-8*β* with H-6*β*, H_3_-14 and H_3_-12(13). Combined with the positive optical rotation, compound **3** was established as methyl 11-hydroxyeudesm-4-en-7*α*H-15-oate.

The known compounds were identified as 11*α*,13-dihydroyomogin (**2**) [31], 1*β*,6*α*-dihydroxyeudesm-4(15)-ene (**4**) [32], 4-*epi*-vulgarin (**5**) [33], ludartin (**6**) [34], dehydroleucodin (**7**) [35], matricarin (**8**) [36], (7R*)-5-*epi*-opposit-4(15)-ene-1*β*,7-diol (**9**) [37], *cis*-hydroxydavanone (**10**) [38], (6*S*,7*S*,l0*R*)-2-hydroperoxy-2,6,10-trimethyl-7,10-epoxydodeca-3,11-dien-5-one (**11**) [39], 11-hydroxy-8-oxo-9,10-dehydro-10,11-dihydronerolidol (**12**) [40], 4-hydroxyacetophenone (**13**) [41], espeletone (**14**) [42], (*S*)-3-(1-hydroxy-3-methylbutyl)-4-methoxyacetophenone (**15**) [43], (*R*)-1-[5-(1-hydroxyethyl)-2-methoxyphenyl]-3-methyl-1-butanone (**16**) [44], and 6-acetyl-3,7-dihydroxy-2,2-dimethyl-chromene (**17**) [45] by comparison of their spectroscopic data with those previously reported.

Compounds **1–17** were evaluated for their cytotoxicity against HSC-LX2. Twelve compounds showed activities with inhibitory ratios higher than 50% at 400 *μ*M (Table 2) and IC_50_ values ranging from 35.1 to 370.3 *μ*M (Table 3). Compounds **2**, **7**, **10–12** and **17** displayed potent cytotoxicity with IC_50_ values of 82.1, 35.1, 95.0, 83.8, 81.6 and 90.1 *μ*M, indicating more potent cytotoxicity than the positive control silybin (IC_50_, 169.6 *μ*M); compound **14** exhibited moderate cytotoxicity with the IC_50_ value of 151.7 *μ*M; compounds **1**, **3**, **5**, **6** and **15** demonstrated less effective than silybin with the IC_50_ values in the range of 204.9**–**370.3 *μ*M. In order to better comprehend the cytotoxic mechanism, the effects of active compounds on the deposition of Col I, HA and HL in HSC-LX2 were assayed by ELISA analysis. Notably, compound **7** as the most active one against HSC-LX2 showed obvious inhibitory effects on the secretion of Col I, HA and HL with IC_50_ values of 10.7, 24.5 and 13.3 *μ*M, which were significantly superior to silybin with IC_50_ values of 54.3, 228.4 and 89.9 *μ*M (Table 4).

**Table 2:** Inhibitory ratios of compounds **1**–**17** on HSC-LX2 at 400 *μ*M

Compound	Inhibitory ratio (%)^a^	Compound	Inhibitory ratio (%)^a^
**1**	77.9 ± 1.0	**10**	101.3 ± 0.5
**2**	79.8 ± 1.1	**11**	101.4 ± 0.6
**3**	74.2 ± 3.6	**12**	102.9 ± 1.4
**4**	48.7 ± 10.1	**13**	30.0 ± 2.5
**5**	54.8 ± 3.5	**14**	76.0 ± 4.3
**6**	89.5 ± 0.6	**15**	64.2 ± 1.0
**7**	102.2 ± 0.3	**16**	47.3 ± 4.0
**8**	49.3 ± 3.9	**17**	77.4 ± 1.2
**9**	48.7 ± 1.9	Silybin^b^	76.7 ± 6.9

^a^Data were expressed as means ± SD (n = 3)

^b^Silybin was used as the positive control

**Table 3:** IC_50_ values of compounds against HSC-LX2

Compound	IC_50_ (*μ*M)^a^	Compound	IC_50_ (*μ*M)^a^
**1**	218.7 ± 4.0	**11**	83.8 ± 1.3
**2**	82.1 ± 7.2	**12**	81.6 ± 3.3
**3**	267.7 ± 8.2	**14**	151.7 ± 7.5
**5**	370.3 ± 23.6	**15**	219.9 ± 15.6
**6**	204.9 ± 4.0	**17**	90.1 ± 9.2
**7**	35.1 ± 0.3	Silybin^b^	169.6 ± 3.4
**10**	95.0 ± 6.9		

^a^Data were expressed as means ± SD (n = 3)

^b^Silybin was used as the positive control

**Table 4:** Inhibitory effect of compound **7** on Col I, HA and HL deposition in HSC-LX2

Compound	IC_50_ (*μ*M)^a^		
	Col I	HA	HL
**7**	10.7 ± 2.5	24.5 ± 0.5	13.3 ± 1.6
Silybin^b^	54.3 ± 9.3	228.4 ± 19.4	89.9 ± 7.2

^a^Data were expressed as means ± SD (n = 3)

^b^Silybin was used as the positive control

## Conclusion

In conclusion, the phytochemical investigations on *A. lavandulaefolia* revealed two new sesquiterpenoids and fifteen known compounds, of which artemlavanin A (**1**) was a rearranged eudesmanolide with an aromatic ring and artemlavanin B (**3**) was a methyl ester derivative of (+)-*γ-*eudesmol. In addition, the chemical constituents isolated from *A. lavandulaefolia* were composed of eudesmane (**2**, **4** and **5**), guaiane (**6–8**), oppositane (**9**) and farnesane (**10–12**) sesquiterpenoids, together with the phenyl derivatives with a 4-hydroxyacetophenone moiety (**13–17**). Compounds **2**, **7**, **10–12** and **17** showed obvious cytotoxicity against HSC-LX2 with IC_50_ values of 82.1, 35.1, 95.0, 83.8, 81.6 and 90.1 *μ*M. Interestingly, compound **7** possessed inhibitory activity on not only HSC-LX2 but also the accumulation of Col I, HA and HL with IC_50_ values of 10.7, 24.5 and 13.3 *μ*M. These results provided the evidence for the antihepatic fibrosis potential of *A. lavandulaefolia*.

## Experimental Section

### General Experimental Procedures

Optical rotations were measured on an Autopol VI automatic polarimeter (Rudolph Research Analytical, Hackettstown, NJ, USA). UV spectra were recorded on a Shimadzu UV2401PC spectrophotometer (Shimadzu, Kyoto, Japan). IR spectra were determined on a NICOLET iS10 (Thermo scientific, Madison, USA) spectrometers using ATR ITX-DIAMOND mode. HRESIMS data were acquired on an LCMS-IT-TOF mass spectrometer (Shimadzu, Kyoto, Japan). 1D and 2D NMR data were obtained on Avance III-600 spectrometers (Bruker, Bremerhaven, Germany). ECD spectra were collected on an Applied Photophysics Chirascan spectrometer (Applied Photophysics, Surrey, UK). Silica gel (200–300 mesh, Qingdao Makall group Co., Ltd., Qingdao, China) and Sephadex LH-20 (GE Healthcare Amersham Biosciences, Uppsala, Sweden) were used for column chromatography. MPLC separations was conducted on a Dr-Flash II apparatus (Lisure Science Co., Ltd., Suzhou, China) using MCI gel CHP 20P column (75–150 *μ*m, Mitsubishi Chemical Corporation, Tokyo, Japan). Semipreparative HPLC separations were performed on a Shimadzu LC-CBM-20 system (Shimadzu, Kyoto, Japan) with an Agilent Eclipse XDB-C_18_ column (5 *μ*m, 9.4 × 250 mm, Agilent Technologies, Santa Clara, USA) unless otherwise specified. TLC analyses were carried out using silica gel GF_254_ plates (Jiangyou Silica Gel Development Co., Ltd., Yantai, China), and compounds were detected by heating after spraying with 10% H_2_SO_4_ in EtOH. Hepatic stellate cell line LX2 (Jining Industrial Co., Ltd., Shanghai, China) were maintained in RPMI 1640 medium supplemented with 10% fetal bovine serum (Gibco BRL, NY, USA). The cytotoxicity was assessed by MTT (BioFROXX, Guangzhou, China) method. The ELISA kits (Cusabio Biotech Co., Ltd., Wuhan, China) was used for the detection of the human collagen type I (Col I) and human hyaluronic acid (HA), and the ELISA kit (Boster Biological Technology Co., Ltd., California, USA) was applied to determine the human laminin (HL). The absorbance was measured on a microplate reader (Bio-Rad, Hercules, CA, USA).

### Plant Materials

The aerial parts of *Artemisia lavandulaefolia* DC. were collected in October, 2018, from Dali, Yunnan Province, China, and authenticated by Prof. Dr. Li-Gong Lei (Key Laboratory of Biodiversity and Biogeography, Kunming Institute of Botany, Chinese Academy of Sciences). A voucher specimen (No. 20181104) was deposited at the Laboratory of Anti-virus and Natural Medicinal Chemistry, Kunming Institute of Botany, Chinese Academy of Sciences.

### Extraction and Isolation

The air-dried aerial parts of *Artemisia lavandulaefolia* (20 kg) were powdered and extracted with 95% EtOH for two times (2 × 100 L × 4 day) at room temperature. The combined extract was concentrated under reduced pressure to yield the residue, which was suspended in H_2_O and subsequently partitioned with EtOAc. The EtOAc fraction (814 g) was subjected to silica gel column chromatography (CC) using EtOAc**–**petroleum ether (PE) gradient (0:100, 5:95, 10:90, 20:80, 40:60 and 100:0, v/v) to give fractions A**–**F (122, 75, 87, 34, 91 and 363 g).

Fraction C (87 g) was separated by silica gel CC with Me_2_CO**–**PE gradient (5:95, 10:90 and 20:80) as the eluent to give fractions C1**–**C3 (36, 35 and 10 g). Fraction C1 was submitted on MPLC eluted with H_2_O**–**MeOH gradient (10:90 and 0:100) to give two fractions (Frs. C1.1 and C1.2). Compounds **1** (12 mg, *t*_R_ = 31 min) and **14** (37 mg, *t*_R_ = 26 min) was obtained from fraction C1.1 by repeated silica gel CC (EtOAc**–**PE, 10:90) and semipreparative HPLC (H_2_O**–**MeCN, 56:44). Fraction C2 was purified by MPLC using H_2_O**–**MeOH gradient (50:50, 30:70, 10:90 and 0:100) to give four fractions (Frs. C2.1**–**C2.4). Fraction C2.2 was loaded on a silica gel CC and eluted with EtOAc**–**PE gradient (5:95, 10:90 and 20:80) to yield three fractions (Frs. C2.2.1**–**C2.2.3). Fraction C2.2.1 was separated by silica gel CC (EtOAc**–**CHCl_3_, 2:98) to afford compounds **10** (296 mg) and **11** (753 mg), and subsequently purified by semipreparative HPLC (H_2_O**–**MeCN, 67:33) to give compounds **4** (85 mg, *t*_R_ = 26 min) and **12** (10 mg, *t*_R_ = 29 min). Fraction C2.3 was chromatographed on a silica gel CC and eluted with Me_2_CO**–**PE (5:95 and 10:90) to provide three fractions (Frs. C2.3.1**–**C2.3.3). Fraction C2.3.1 was carried out on silica gel CC and eluted with PE**–**CHCl_3_ gradient (1:99 and 2:98) to produce two fractions (Frs. C2.3.1.1 and C2.3.1.2). Compounds **15** (8 mg, *t*_R_ = 50 min) and **16** (9 mg, *t*_R_ = 54 min) were obtained from fraction C2.3.1.1 by semipreparative HPLC (H_2_O**–**MeCN, 73:27) using Opti-Chiral® C1-5 (RP) column (5 *μ*m, 10 × 250 mm, ColumnTek, LLC, 200 Innovation Blvd., Suite 258A, State College, PA 16803 USA). Fraction C2.3.1.2 was chromatographed on a Sephadex LH-20 CC (MeOH**–**CHCl_3_, 50:50) to provide compound **13** (10 mg) and subsequently purified by semipreparative HPLC (H_2_O**–**MeCN, 53:47) to yield compound **17** (189 mg, *t*_R_ = 14 min). Compounds **3** (25 mg), **6** (57 mg) and **7** (16 mg) were obtained from fraction C2.3.2 by repeated silica gel CC (EtOAc**–**PE, 3:97 and 10:90) and Sephadex LH-20 CC (MeOH**–**CHCl_3_, 50:50). Fraction C3 was separated by MPLC using H_2_O**–**MeOH gradient (30:70, 10:90 and 0:100) to generate three fractions (Frs. C3.1**–**C3.3). Compound **9** (10 mg) was isolated from fraction C3.1 by repeated silica gel CC (EtOAc**–**PE, 10:90) and Sephadex LH-20 CC (MeOH**–**CHCl_3_, 50:50).

Fraction D (34 g) was subjected to MPLC and eluted with H_2_O**–**MeOH gradient (40:60, 60:40, 20:80, and 0:100) to yield fractions D1**–**D4 (0.9, 6.8, 9.0 and 11 g). Fraction D3 was separated by silica gel CC eluted with EtOAc**–**PE (20:80) and semipreparative HPLC (H_2_O**–**MeCN, 70:30) to give compounds **5** (57 mg, *t*_R_ = 18 min) and **8** (31 mg, *t*_R_ = 34 min). Fraction D4 was chromatographed on a silica gel CC using EtOAc**–**CHCl_3_ as eluent (5:95) to afford two fractions (Frs. D4.1 and D4.2). Compound **2** (46 mg, *t*_R_ = 18 min) was obtained from fraction D4.1 by semipreparative HPLC (H_2_O**–**MeCN, 70:30).

Artemlavanin A (**1**): colorless oil; [*α*]_D_^22^ + 15.2 (*c* 0.10, MeOH); UV (MeOH) *λ*_max_ (log *ε*) 220 (3.91) nm; ECD (MeOH) *λ*_max_ (Δ*ε*) 205 (+ 43.2) nm; IR *v*_max_ 1752, 1488, 1454, 1189 cm^−1^; ^1^H NMR and ^13^C NMR data see Table 1; (+)-HRESIMS *m/z* 231.1383 [M+H]^+^ (calcd for C_15_H_19_O_2_, 231.1380).

Artemlavanin B (**3**): colorless oil; [*α*]_D_^20^ + 68.9 (*c* 0.10, MeOH); UV (MeOH) *λ*_max_ (log *ε*) 225 (3.77) nm; IR *v*_max_ 3455, 1713, 1625, 1241, 1218 cm^−1^; ^1^H NMR and ^13^C NMR data see Table 1; (+)-HRESIMS *m/z* 289.1754 [M+Na]^+^ (calcd for C_16_H_26_O_3_Na, 289.1774).

### Cytotoxicity Assay

The cytotoxicity of compounds was determined using the MTT method. Briefly, HSC-LX2 were cultured in 96-well plates at a density of 1 × 10^4^ cells/well in RPMI 1640 medium supplemented with 10% fetal bovine serum at 37 ℃ with 5% CO_2_. After overnight incubation, cells were treated with compounds at different concentrations for 48 h. Subsequently, the culture medium was exchanged by 100 *μ*L of MTT reagent (1 mg/mL). After co-incubation for 4 h at 37 °C, the solution was removed and 100 *μ*L of DMSO was added to dissolve the formazan crystals. The absorbance was recorded on a microplate reader at 490 nm. All the experiments were carried out in triplicate. The inhibitory ratios were calculated as [A_(control)_−A_(sample)_]/A_(control)_ × 100%, and IC_50_ values were calculated using GraphPad Prism 5 (GraphPad Software, San Diego, CA, USA).

### Col I, HA and HL Secretion Assay

HSC-LX2 were seeded at 8 × 10^4^ cells/well in 24-well plates overnight and then treated with the compounds at different concentrations. After 72 h incubation, Col I, HA and HL levels of culture media were collected and determined using the commercial ELISA kits according to the manufacturer's instructions.

## Electronic supplementary material

Below is the link to the electronic supplementary material.

Supplementary file1 (DOCX 2497 kb)
