# Supplementary material for: Artemlavanins A and B from Artemisia lavandulaefolia and Their Cytotoxicity Against Hepatic Stellate Cell Line LX2
Source: Nat Prod Bioprospect. 2020 Jun 24;10(4):243–50. doi: 10.1007/s13659-020-00254-0 (PMC7367947; doi:10.1007/s13659-020-00254-0)
Supplement: Supplementary file 1 — Supplementary file1 (DOCX 2497 kb) [file 13659_2020_254_MOESM1_ESM.docx]

**Supporting Information**

**Artemlavanins A and B from *Artemisia lavandulaefolia* and Their Cytotoxicity against** **Hepatic Stellate Cells Line LX2**

**Cheng Shen^1,2,3^ · Xiao-Yan Huang^1,2^ · Chang-An Geng^1,2^ · Tian-Ze Li^1,2^ · Shuang Tang^1,2,3^ · Li-Hua Su^1,2,3^ · Zhen Gao^1,2,3^ · Xue-Mei Zhang^1,2^ · Jing Hu^1,2^ · Ji-Jun Chen^1,2,3,*^**

^1^ State Key Laboratory of Phytochemistry and Plant Resources in West China, Kunming Institute of Botany, Chinese Academy of Sciences, Kunming 650201, People's Republic of China

^2^ Yunnan Key Laboratory of Natural Medicinal Chemistry, Kunming 650201, People's Republic of China

^3^ University of Chinese Academy of Sciences, Beijing 100049, People's Republic of China

Corresponding Author

*Tel: + 86-871-65223265. Fax: +86-871-65227197. E-mail: chenjj@mail.kib.ac.cn

Contents

[**Figure S1.** HRESIMS spectrum of compound **1** 3](#_Toc40650914)

[**Figure** **S2.** [*α*]_D_ spectrum of compound **1** in MeOH 3](#_Toc40650915)

[**Figure S3.** IR spectrum of compound **1** 4](#_Toc40650916)

[**Figure** **S4.** UV spectrum of compound **1** 4](#_Toc40650917)

[**Figure S5.** ECD spectrum of compound **1** in MeOH 5](#_Toc40650918)

[**Figure** **S6.** ^1^H NMR spectrum of compound **1** (600 MHz, CDCl_3_) 5](#_Toc40650919)

[**Figure S7.** ^13^C NMR (DEPT) spectrum of compound **1** (150 MHz, CDCl_3_) 6](#_Toc40650920)

[**Figure S8.** HSQC spectrum of compound **1** 6](#_Toc40650921)

[**Figure S9.** HMBC spectrum of compound **1** 7](#_Toc40650922)

[**Figure** **S10.** ^1^H–^1^H COSY spectrum of compound **1** 7](#_Toc40650923)

[**Figure S11.** ROESY spectrum of compound **1** 8](#_Toc40650924)

[**Figure** **S12.** HRESIMS spectrum of compound **3** 9](#_Toc40650925)

[**Figure** **S13.** [*α*]_D_ spectrum of compound **3** in MeOH 9](#_Toc40650926)

[**Figure S14.** IR spectrum of compound **3** 10](#_Toc40650927)

[**Figure S15.** UV spectrum of compound **3** 10](#_Toc40650928)

[**Figure** **S16.** ^1^H NMR spectrum of compound **3** (600 MHz, CDCl_3_) 11](#_Toc40650929)

[**Figure** **S17.** ^13^C NMR (DEPT) spectrum of compound **3** (150 MHz, CDCl_3_) 11](#_Toc40650930)

[**Figure S18.** HSQC spectrum of compound **3** 12](#_Toc40650931)

[**Figure** **S19.** HMBC spectrum of compound **3** 12](#_Toc40650932)

[**Figure S20.** ^1^H–^1^H COSY spectrum of compound **3** 13](#_Toc40650933)

[**Figure S21.** ROESY spectrum of compound **3** 13](#_Toc40650934)

[Computational details 14](#_Toc40650935)


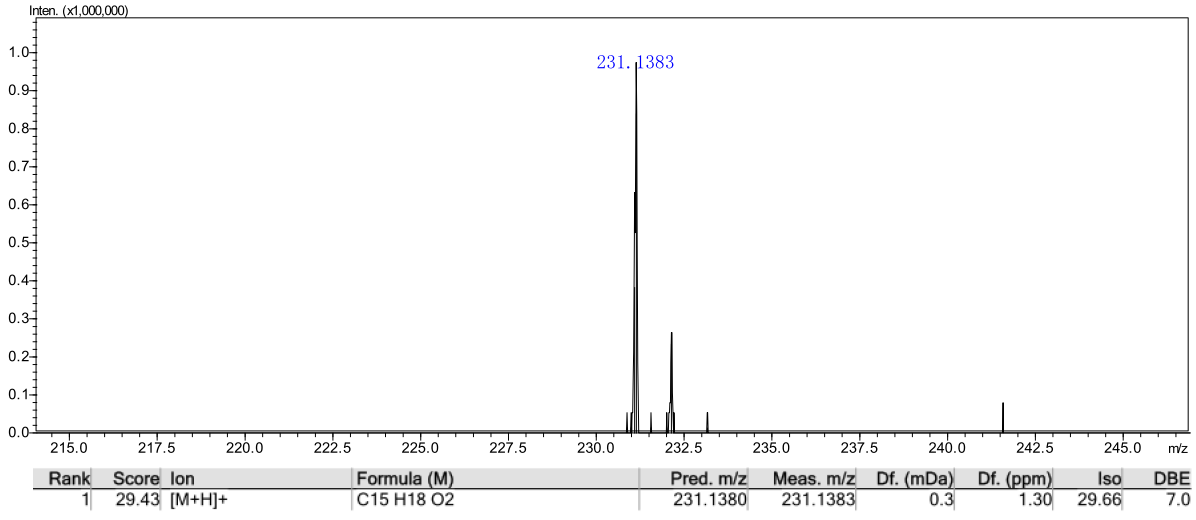


# Figure S1. HRESIMS spectrum of compound 1


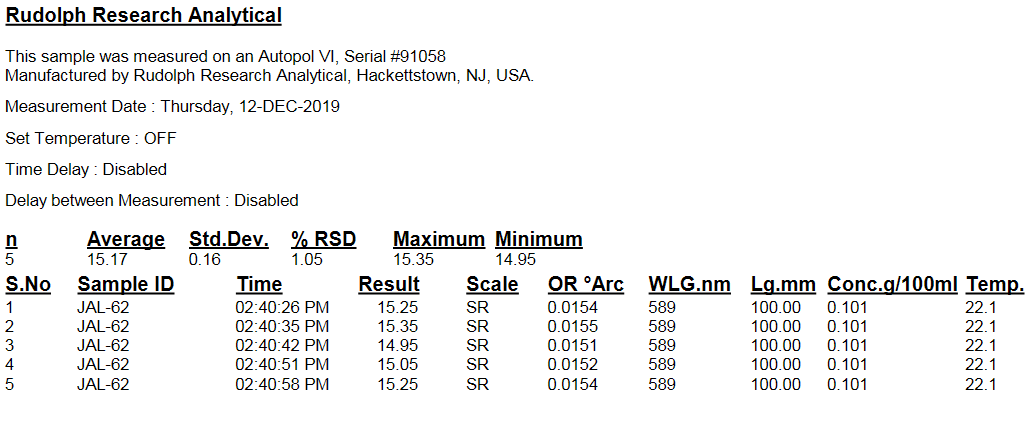


# Figure S2. [*α*]_D_ spectrum of compound 1 in MeOH


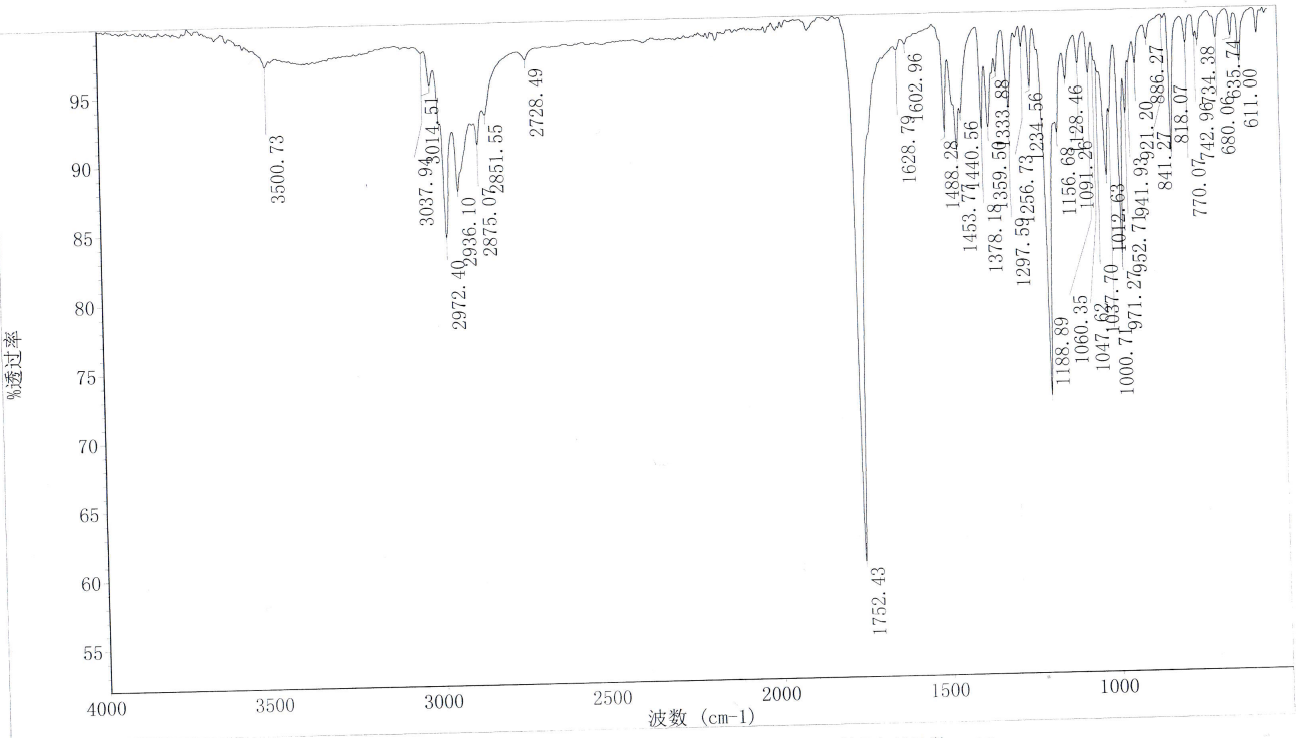


# Figure S3. IR spectrum of compound 1


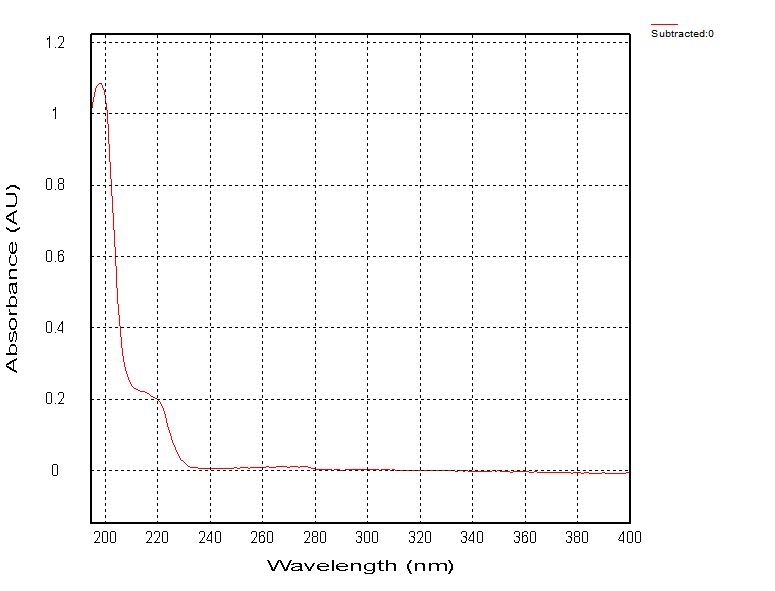


# Figure S4. UV spectrum of compound 1


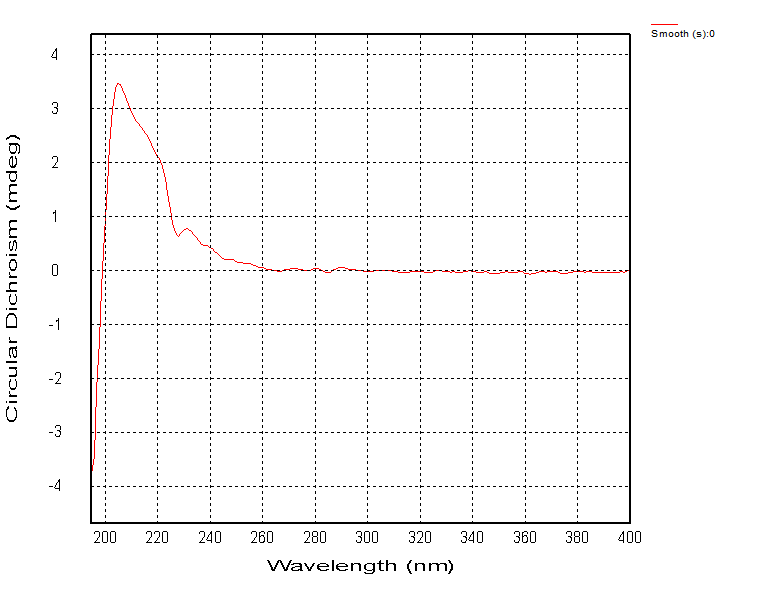


# Figure S5. ECD spectrum of compound 1 in MeOH

# Figure S6. ^1^H NMR spectrum of compound 1 (600 MHz, CDCl_3_)

# Figure S7. ^13^C NMR (DEPT) spectrum of compound 1 (150 MHz, CDCl_3_)

# Figure S8. HSQC spectrum of compound 1

# Figure S9. HMBC spectrum of compound 1

# Figure S10. ^1^H–^1^H COSY spectrum of compound 1

# Figure S11. ROESY spectrum of compound 1


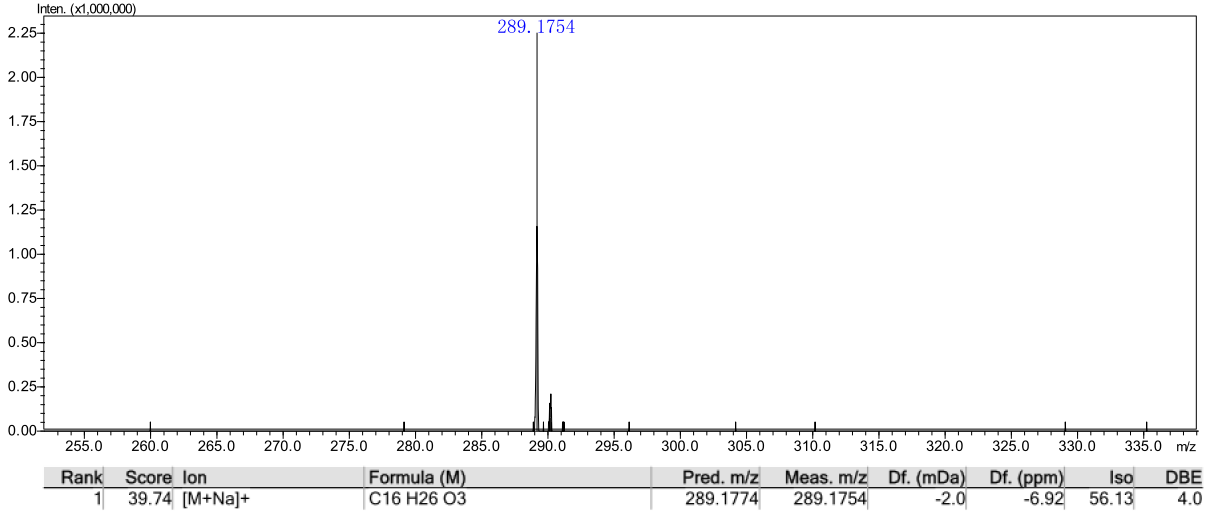


# Figure S12. HRESIMS spectrum of compound 3


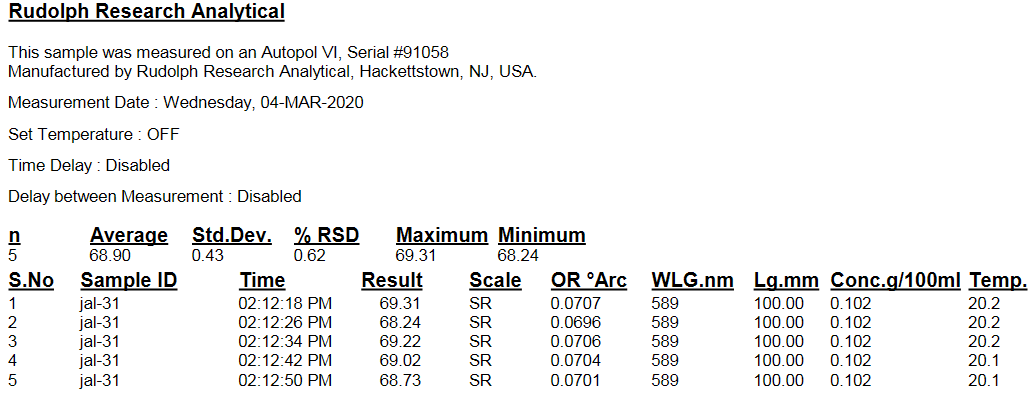


# Figure S13. [*α*]_D_ spectrum of compound 3 in MeOH


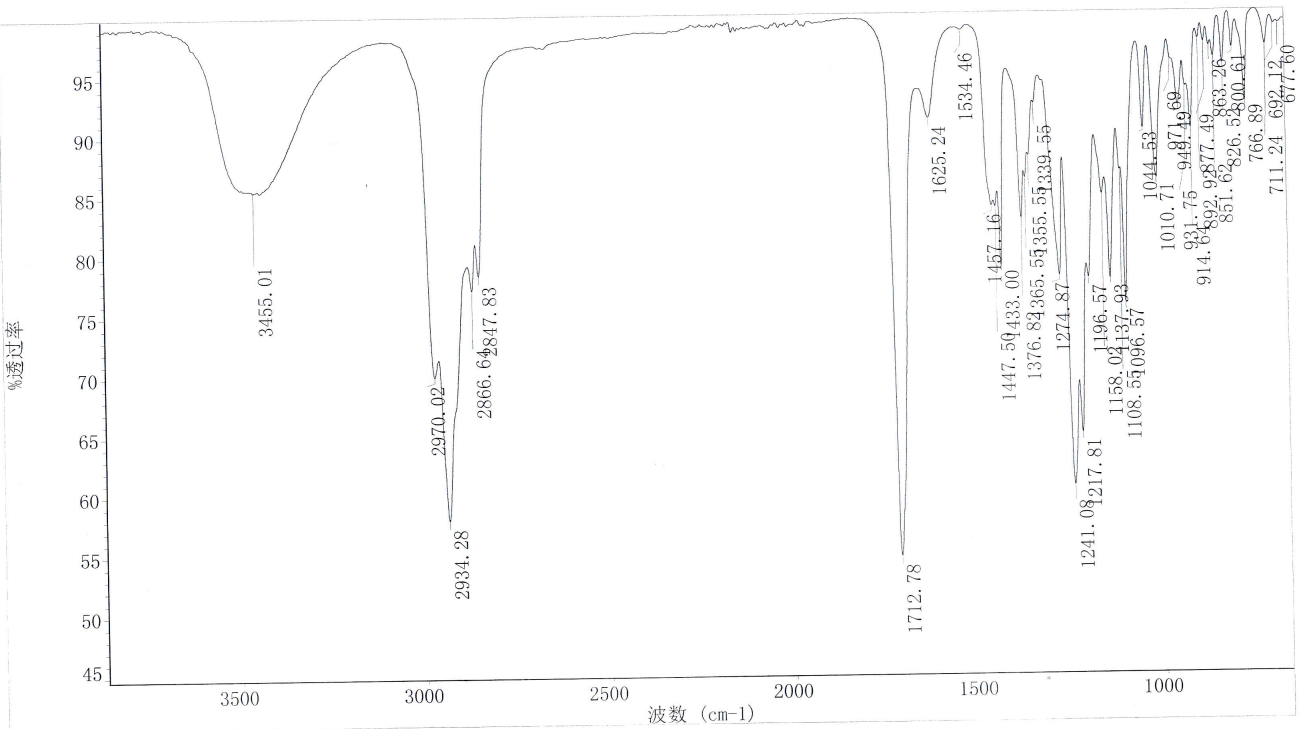


# Figure S14. IR spectrum of compound 3


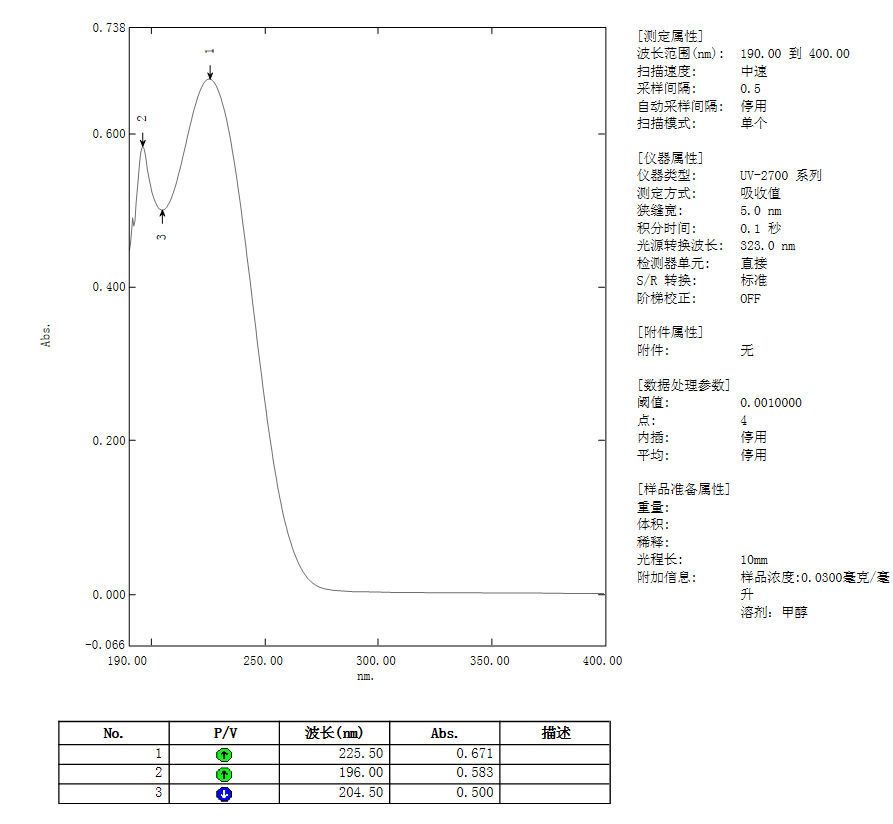


# Figure S15. UV spectrum of compound 3

# Figure S16. ^1^H NMR spectrum of compound 3 (600 MHz, CDCl_3_)

# Figure S17. ^13^C NMR (DEPT) spectrum of compound 3 (150 MHz, CDCl_3_)

# Figure S18. HSQC spectrum of compound 3

# Figure S19. HMBC spectrum of compound 3

# Figure S20. ^1^H–^1^H COSY spectrum of compound 3

# Figure S21. ROESY spectrum of compound 3

# Computational details

Conformation search based on molecular mechanics with MMFF94S force fields were performed and gave corresponding stable conformers. All the conformers with distributions higher than 1% were further optimized by means of the DFT calculation at b3lyp/6-31G(d,p) level in the gas phase. The imaginary frequencies were excluded by the aid of frequency calculations at the same level. ECD calculation was performed using time-dependent density-functional theory (TDDFT) at b3lyp/6-311+g(d,p) level in MeOH using PCM model. The calculated ECD data were Boltzmann-averaged based on Gibbs free energy. All the DFT calculations were performed with the Gaussian09 software package and the ECD spectra were simulated by the SpecDis program [1].

1. M. J. Frisch, G. W. Trucks, H. B. Schlegel, G. E. Scuseria, M. A. Robb, J. R. Cheeseman, G. Scalmani, V. Barone, B. Mennucci, G. A. Petersson, H. Nakatsuji, M. Caricato, X. Li, H. P. Hratchian, A. F. Izmaylov, J. Bloino, G. Zheng, J. L. Sonnenberg, M. Hada, M. Ehara, K. Toyota, R. Fukuda, J. Hasegawa, M. Ishida, T. Nakajima, Y. Honda, O. Kitao, H. Nakai, T. Vreven, J. A. Montgomery, Jr., J. E. Peralta, F. Ogliaro, M. Bearpark, J. J. Heyd, E. Brothers, K. N. Kudin, V. N. Staroverov, T. Keith, R. Kobayashi, J. Normand, K. Raghavachari, A. Rendell, J. C. Burant, S. S. Iyengar, J. Tomasi, M. Cossi, N. Rega, J. M. Millam, M. Klene, J. E. Knox, J. B. Cross, V. Bakken, C. Adamo, J. Jaramillo, R. Gomperts, R. E. Stratmann, O. Yazyev, A. J. Austin, R. Cammi, C. Pomelli, J. W. Ochterski, R. L. Martin, K. Morokuma, V. G. Zakrzewski, G. A. Voth, P. Salvador, J. J. Dannenberg, S. Dapprich, A. D. Daniels, O. Farkas, J. B. Foresman, J. V. Ortiz, J. Cioslowski, D. J. Fox, Gaussian 09, Revision C.01; Gaussian, Inc., Wallingford CT: 2010.
